# Reamed intramedullary nailing versus circular frame external fixation for segmental tibial fractures (STIFF-F): a mixed methods feasibility study

**DOI:** 10.1186/s40814-021-00821-3

**Published:** 2021-04-10

**Authors:** Caroline B. Hing, Elizabeth Tutton, Toby O. Smith, Molly Glaze, Jamie R. Law, Jonathan Cook, Melina Dritsaki, Emma Phelps, Cushla Cooper, Alex Trompeter, Michael Pearse, Michael Law, Matthew L. Costa

**Affiliations:** 1 Department of Trauma and Orthopaedics, St George’s University Hospitals NHS Foundation Trust, London, UK; 2 Nuffield Department of Orthopaedics, Rheumatology and Musculoskeletal Sciences, University of Oxford, Oxford, UK; 3 Faculty of Medicine and Health Sciences, University of East Anglia, Norwich, Norfolk, UK; 4 Imperial College London, London, UK

**Keywords:** Equipoise, Feasibility, Randomised controlled trial, Qualitative, Interviews, Survey

## Abstract

**Background:**

Segmental tibial fractures are fractures in two or more areas of the tibial diaphysis resulting in a separate intercalary segment of the bone. Surgical fixation is recommended for patients with segmental tibial fractures as non-operative treatment outcomes are poor. The most common surgical interventions are intramedullary nailing (IMN) and circular frame external fixation (CFEF), but evidence about which is better is of poor quality. An adequately powered randomised controlled trial (RCT) to determine optimum treatment is required. STIFF-F aimed to assess the feasibility of a multicentre RCT comparing IMN with CFEF for segmental tibial fracture.

**Methods:**

STIFF-F was a mixed-methods feasibility study comprising a pilot RCT conducted at six UK Major Trauma Centres, qualitative interviews drawing on Phenomenology and an online survey of rehabilitation. The primary outcome was recruitment rate. Patients, 16 years and over, with a segmental tibial fracture (open or closed) deemed suitable for IMN or CFEF were eligible to participate. Randomisation was stratified by site using random permuted blocks of varying sizes. Participant or assessor blinding was not possible. Interviews were undertaken with patients about their experience of injury, treatment, recovery and participation. Staff were interviewed to identify contextual factors affecting trial processes, their experience of recruitment and the treatment pathway. An online survey was developed to understand the rehabilitation context of the treatments.

**Results:**

Eleven patients were screened and three recruited to the pilot RCT. Nineteen staff and four patients participated in interviews, and 11 physiotherapists responded to the survey. This study found the following: (i) segmental tibial fractures were rarer than anticipated, (ii) the complexity of the injury, study setup times and surgeon treatment preferences impeded recruitment, (iii) recovery from a segmental tibial fracture is challenging, and rehabilitation protocols are inconsistent and (iv) despite the difficulty recruiting, staff valued this research question and strived to find a way forward.

**Conclusion:**

The proposed multicentre RCT comparing IMN with CFEF is not feasible. This study highlighted the difficulty of recruiting patients to an RCT of a complex rare injury over a short time period.

**Trial registration:**

The study was registered with the International Standard Randomised Controlled Trials Number Registry: ISRCTN11229660

## Key messages regarding feasibility

- What uncertainties existed regarding the feasibility? RCTs of surgical interventions for rare conditions are challenging as both surgeons and patients have strong preferences for particular treatments. A feasibility study is needed to determine whether surgeons and patients will participate in a trial of two treatments for segmental tibial fractures.
- What are the key feasibility findings? Due to the rarity and complexity of segmental tibial fractures, an RCT comparing IMN and CFEF is not feasible.
- What are the implications of the feasibility findings for the design of the main study? Alternative study designs should be considered to aid understanding of the best way to treat and improve outcomes for patients with rare complex injuries including segmental tibial fractures.

## Background

Segmental tibial fractures are rare fractures of the tibial diaphysis resulting in a separate intercalary segment of the bone. Their severity means treatment outcomes are often poor, and patients are at risk of amputation if treatment fails [1]. Patients suffer greatly as they struggle with pain, immobility and body image [2–5].

**Fig. 1:**
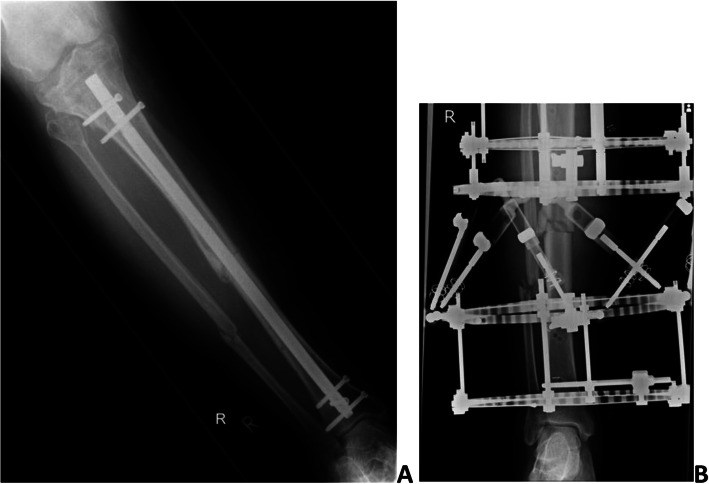
**a** Radiograph showing segmental tibial fracture fixation with IMN. **b** Radiograph showing CFEF fixation

Surgical fixation is recommended for all segmental tibial fracture patients as non-operative treatment outcomes are poor [6]. Commonly used surgical options include intramedullary nailing (IMN) or circular frame external fixation (CFEF) [7]. IMN involves reducing the fracture, inserting a metal rod down the centre of the bone and fixing it in position with screws. No metalwork is visible outside of the skin, and the metal rod stays permanently within the bone (Fig. 1a, radiograph showing segmental tibial fracture fixation with IMN). CFEFs form an external scaffold, pulling the fracture fragments together. The frame remains in place until the bone has healed, but at the end of treatment, no metal is left in the body (Fig. 1b, radiograph showing CFEF fixation). Evidence comparing IMN and CFEF is of low-quality, based largely on poorly matched cohort studies where patient-reported outcome measures are rarely used [7]. The clinical consensus for treating these fractures (between IMN and CFEF) is low; however, there was enough evidence of equipoise within the orthopaedic community [8] to support this feasibility study.

Recruitment to trials of surgical interventions are known to be challenging due to complex patient pathways, variation in surgeons’ understanding of research protocols, limited surgical equipoise and patients’ reservations about recruitment [9–11]. In trauma trials, patients can feel their treatment is compromised and feel they lack sufficient information to make informed choices [11, 12]. In light of these challenges, STIFF-F aimed to assess the feasibility of a multicentre randomised controlled trial (RCT) comparing IMN with CFEF*.*

## Methods

STIFF-F was a mixed-methods feasibility study comprising a pilot RCT, qualitative interviews and an online rehabilitation survey. The protocol for this study is available in the online supplementary material.

### Pilot randomised trial

**Fig. 2:**
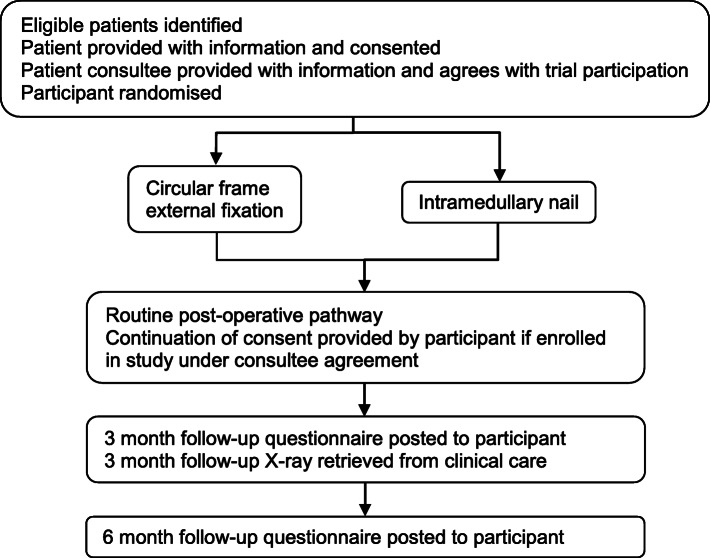
Patient flow diagram for randomised pilot within STIFF-F

The pilot study aimed to randomise 50 patients to receive either IMN or CFEF in a 1:1 ratio, with 6 months follow-up (Fig. 2, patient flow diagram for randomised pilot within STIFF-F). Adults with a segmental tibial fracture (open or closed) deemed suitable for either intervention were screened at UK Major Trauma Centres (MTCs). Exclusions included the following parameters: under 16 years, prior failed fixation, pathological fracture, infection, pre-existing skin condition which precluded open surgery, more than 21 days since injury, and patients who would be unable to understand treatment instructions regardless of their injury (for example, patients with dementia).

Potential participants were identified in daily trauma meetings. Patients able to give informed consent pre-operatively were consented by the research team. Patients temporarily lacking capacity were entered into the study with consultee agreement. A relative/friend (personal consultee) was informed about the study and asked if in their opinion the patient would object to taking part. If a personal consultee was not available, a nominated consultee (normally a surgical consultant who was not involved in the study but knew the patient) advised about the patient’s participation. The study was discussed with the patient once they regained capacity, who could confirm consent or decline to continue participating.

Both IMN and CFEF were routinely available in all participating sites. The study was pragmatic and perioperative care continued as per the National Health Service (NHS) care provision and local policy. Operative details were collected during participant admission. Questionnaires were sent to participants at 3 and 6 months post-randomisation to complete a disability rating index (DRI), a Pittsburgh Sleep Quality Index (PSQI), a Tampa Scale for Kinesiophobia (TSK), health-related quality of life score (EuroQol EQ-5D-5L) and details of health resource use [13–18]. Radiographs were collected at 3 months to calculate the radiographic union scale in tibial fracture (RUST) score [19].

Randomisation was stratified by site using random permuted blocks of varying sizes. The allocation sequence was generated by the trial statistician, then programmed into the Oxford Clinical Trials Research Unit (OCTRU) randomisation system. The site research teams completed randomisation online via secure log-ins. As the CFEF intervention was clearly visible, no participant or assessor blinding was possible.

### Qualitative interviews

The study drew on phenomenology to develop an understanding of the participants lived experience in light of their social and cultural context [20]. Patients, including those who declined participation in the pilot study, took part in an interview about their experience of injury, treatment, recovery and trial participation. A purposive sample of staff took part in an interview about their experience of participating in the study such as contextual factors affecting trial processes, their experience of recruitment and the treatment pathway. Interviews were lightly structured, conducted face to face or over the telephone in the workplace, by an experienced female qualitative researcher with a background in health sciences and a PhD (ET). Four of the staff participants were known to the researchers prior to the study. Interviews were audio-recorded, transcribed verbatim, took place between November 2019 and April 2020 and were 20–93-min long. Analysis was thematic, with codes, categories and themes of experience identified from interview transcripts (conducted by ET, EP) [21]. NVIVO 11 (QRS International, Warrington, UK) was used to manage the data. In this paper, we present key findings from the qualitative data that relate to the feasibility of a full RCT of IMN and CFEF for segmental tibial fractures. Themes developed from our analysis of staff experiences were saturated, where no new categories and themes are evident in the data. These data will be presented elsewhere. Participants chose not to have a copy of their transcripts.

Several strategies were adopted to ensure rigour [22]. ET and EP were immersed in the data, held regular discussions and reflected upon their positionality throughout analysis. Verbatim quotes are present to illustrate our interpretation of participants’ experience, and detailed description of the context is provided to enable transferability of findings. Resonance with the findings was identified by surgeons, research staff and a PPI representative.

### Rehabilitation survey

To understand the context of patient care for this study, an online survey aimed to determine current rehabilitation and recovery pathways for patients with IMN or CFEF following segmental tibial fractures. The survey (Online surveys, Jisc) was publicised to health professionals involved in the care of patients with segmental tibial fractures, through professional societies (British Orthopaedic Association and Association of Trauma and Orthopaedic Chartered Physiotherapists), pilot site teams and the study teams’ Twitter accounts. Participant consent was implied by completion and submission. The survey covered the start, frequency and nature of postoperative in-patient rehabilitation and discharge destination for patients. The data were analysed descriptively.

## Results

**Fig. 3:**
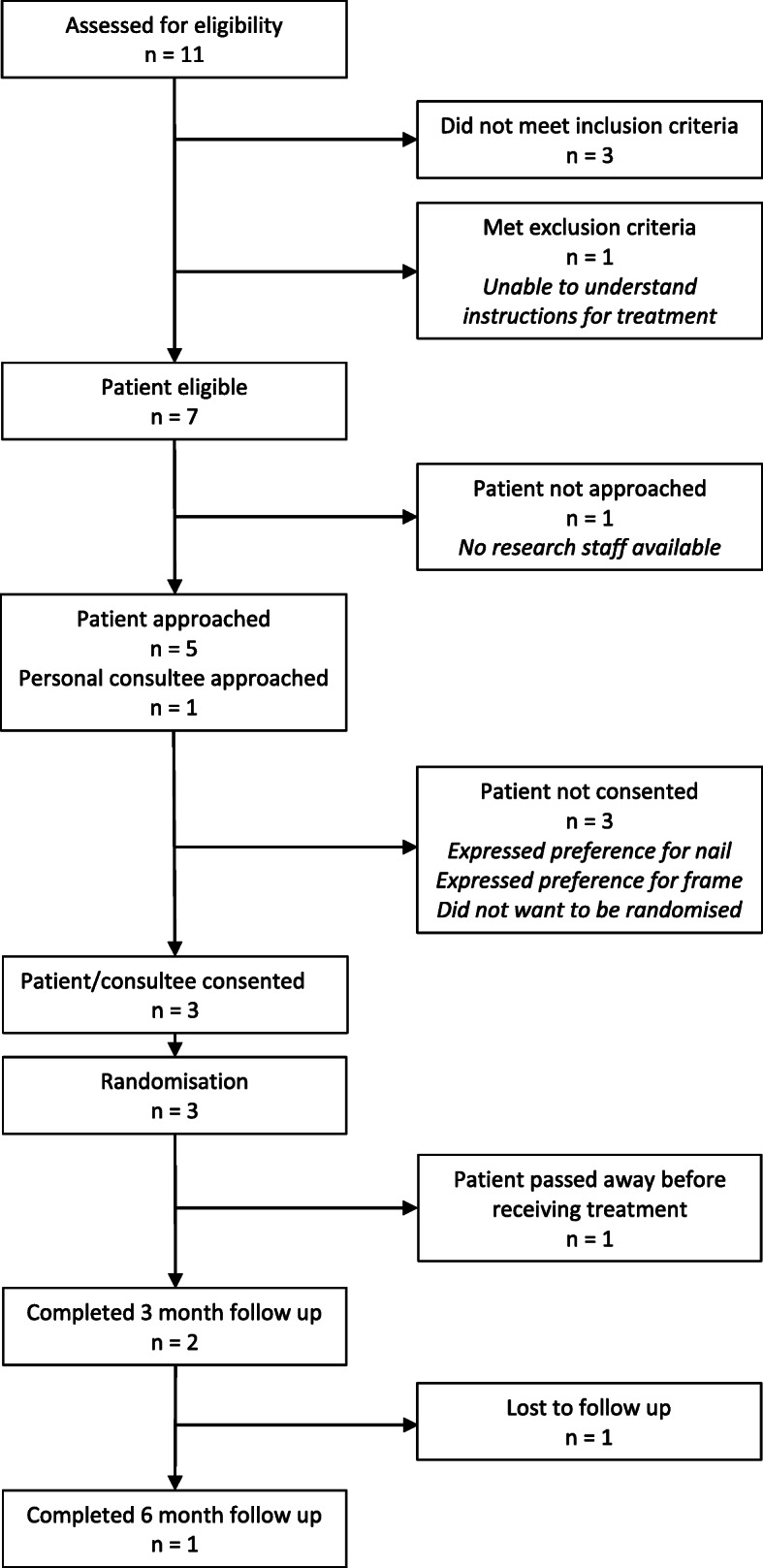
Flow diagram of randomised pilot

The pilot study opened for recruitment in six sites between May 2019 and February 2020. Recruitment was initially planned for 6 months but was extended to account for the delay in setting up sites. The study closed in February 2020 as it was apparent that the recruitment target could not be achieved. During this time, four sites screened 11 patients, and three were recruited to the study (Fig. 3, flow diagram of randomised pilot). Of these, two completed follow-up questionnaires and one died. Patients declined participation due to treatment preferences (*n*=2) or felt randomisation was unacceptable (*n*=1). In addition, three patients did not meet the inclusion criteria, one was excluded as they lacked ability to understand, and for one patient, there were no research staff. Given the low number of participants recruited, no summary statistics were calculated. No adverse events were reported. A purposive sample of nineteen staff and four patients participated in qualitative interviews; no patients or staff declined to take part. Staff included surgeons and research and clinical staff involved in the study. Eleven physiotherapists from 11 hospitals including three MTCs responded to the rehabilitation survey (the number of staff able to respond to the survey is unknown).

### Summary of findings

The findings of this feasibility study show that a multicenter RCT comparing IMN with CFEF is not feasible due to the rarity of the injury. Due to the low recruitment number, not all of the study objectives were met (Table 1, objectives and outcomes for the STIFF-F study). Key findings from this study are as follows:

i) The incidence of segmental tibial fractures was rarer than anticipated,
ii) Several factors including the complexity and severity of injury, lengthy study setup and surgeon treatment preferences inhibited recruitment,
iii) Recovery from a segmental tibial fracture is challenging, and rehabilitation protocols for patients are inconsistent,
iv) This research question is still valued by staff who endeavour to find a way to move forward with it.

**Table 1:** Objectives and outcomes for the STIFF-F study

Objectives	Outcomes	Summary of feasibility findings
Primary objective: Assess the feasibility of a multicentre RCT comparing IMN with CFEF.	Primary outcome: Rates of recruitment and follow-up in a randomised pilot study.	• Rates of recruitment show that a multicentre RCT is not feasible.
Secondary objectives: Identify conflicts or areas of concern for the research pathway compared with the clinical pathway.	Secondary outcomes: Screening of all adults with segmental tibial fractures, including rationale for eligibility; patient interviews to identify challenges in pathway and factors influencing willingness to consent; staff interviews to determine surgeon and community equipoise, and challenges in the research pathway and culture.	• Sites reported screening patients in daily multi-disciplinary meetings and online systems with little conflict with clinical pathways. Both these methods were well established, and the trauma research teams were experienced. However, there iswas a lack of external data to put the study screening numbers in context. For example, segmental tibial fractures are not reported separately by the Trauma Audit and Research Network. • Three patients declined participation in the study, two due to treatment preferences and one due to a dislike of randomisation. • Surgeon difficulty with equipoise was identified as a barrier to recruitment in this study, with surgeons holding strong preferences based on their beliefs, experience and training.
Assess compliance with the randomised allocation.	Completion of allocated surgical procedure, rationale for all surgical/treatment activity.	• Two patients received their allocated treatment, and 1 died before treatment.
Estimate standard deviation of the outcome measure to estimate the definitive sample size.	Disability Rating Index (function) (DRI) [13].	• This was not estimated due to low recruitment numbers.
Evaluate feasibility of a definitive economic evaluation of IMN versus CFEF.	Health resource use and Health-Related Quality of life (EQ-5D-5L) [14].	• The feasibility study collected data on the use of health care services as well as costs to society in order to capture the overall cost of treatment for segmental tibial fracture. This study has shown that it is possible to collect the data necessary for a full cost-effectiveness analysis of IMNs and CFEFs. There were no practical issues to accessing information for collection and evaluation of the interventions.
Estimate quality of life post-fixation.	Pittsburgh Sleep Quality Index (PSQI) (15), Tampa Scale for Kinesiophobia (fear of movement/injury) (TSK) [16–18] and EQ-5D-5L [14].	• This was not estimated due to low recruitment numbers.
Estimate healing rates.	Radiological images assessed by the Radiographic Union Scale in Tibial fractures (RUST) score [19].	• This was not estimated due to low recruitment numbers.
Review current post-operative rehabilitation regimens.	Rehabilitation survey and interviews with staff and patients to identify current experience of rehabilitation.	• 11 hospitals responded to the survey. • The findings of the survey suggest that protocols to direct rehabilitation for patients with a segmental tibial fracture are lacking. • Interviews with patients and staff reveal that recovery for patients with a segmental tibial fracture is slow and arduous, and support is needed. • Staff perceived patients with CFEF to have greater rehabilitative needs.
Examine variability of patient experiences of injury, treatment and recovery.	Interviews with patients to determine the impact of both treatments and outcomes important to them.	• Patient interviews revealed that recovery was slow and arduous for patients who received both IMN and CFEF.
Explore the views of clinicians and patients on the factors that facilitate or inhibit trial recruitment.	Interviews with staff and patients/consultee to identify the feasibility of undertaking a full trial.	Staff identified the following factors that inhibited recruitment to this study: • Rarity of injury. • Complexity and severity of the injury. • Lack of individual surgeon equipoise. • Surgical skill • Slow site setup and a short recruitment window • Concerns about the pragmatic nature of the trial Staff identified the following factors that could facilitate recruitment to this study: • A mix of surgical skills within the team • Willingness to randomise to expertise • Keen and experienced staff at sites

#### The incidence of segmental tibial fractures was rare

Staff in participating sites reported fewer segmental tibial fractures than anticipated, expressing surprise at the rarity of the injury.

Although we all thought we saw more,… the true segmental diaphyseal fractures we were interested in for the purposes of STIFF-F is actually incredibly rare to the point where, I don’t think, certainly in this centre we’re missing any.

Staff 17 (surgeon)

Staff reported vigilant screening practices and were confident that they were not missing patients. They expressed a need to find all potential patients as they were aware they may not achieve their recruitment target. They screened patients in daily multi-disciplinary meetings and online systems. Both these methods were well-established, and the trauma research teams were experienced. Unfortunately, there is a lack of external data to put the study screening numbers in context. For example, segmental tibial fractures are not reported separately by the Trauma Audit and Research Network.

You do really need to be proactive and kind of question everything and if you see a tibial fracture on the admissions list you’ve got to go into it further, you might be sat in a meeting and it’s discussed and it’s not one, but that’s fine. Because our recruitment numbers were so low, we were all very hyper vigilant, looking out for them, knowing that we weren’t doing particularly well. It wasn’t until a few months later that we realised, that nationally, that people weren’t doing well.

Staff 9 (research staff)

#### Factors inhibiting recruitment to this study

Factors that inhibited recruitment to this study were (a) the clinical complexity of the injury, (b) the short recruitment window and delays getting sites setup and (c) surgeon difficulties with equipoise.

- Staff considered clinical complexity to be a barrier to the STIFF-F study. Major trauma patients with multiple injuries have urgent needs that require intensive care or plastic surgery as a first priority. For example, in STIFF-F, one participant had a personal consultee agreement and was randomised to receive CFEF. They became increasingly unwell and died before fixation. Individual patient factors also needed to be considered. CFEF required cleaning, considerable adjustments to daily living and could be difficult for patients to manage. Surgeons needed to consider whether patients could care for and cope with a CFEF.

The other thing that I would always look at when thinking about patients with frames is, are they actually going to be capable of looking after the frame? Again it might be the right surgical option. However if it’s a person who’s not a great host or has difficult living circumstances or problems with drugs or alcohol, then we would know that their infection rate is going to be a lot higher because of the hygiene and cleaning ability, and we would always look at that.

Staff 16 (clinical staff)

**Fig. 4:**
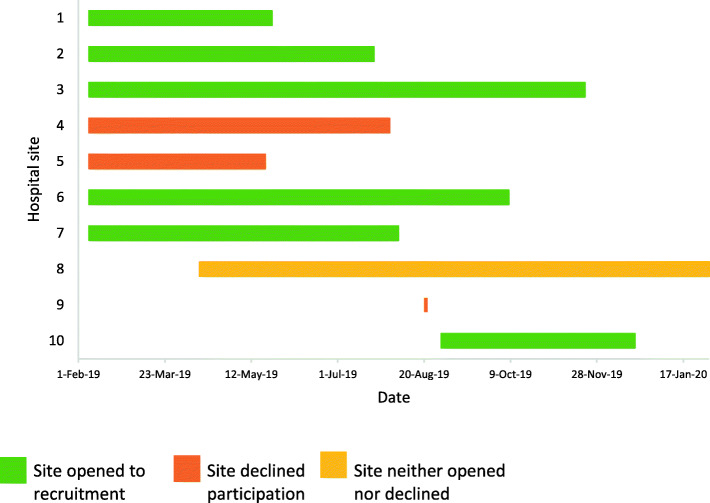
Time taken for sites to open to recruitment or decline participation from initial approach

b) There were delays getting the sites involved and set up. Initially, seven MTCs agreed to take part in the STIFF-F pilot. With only a 6-month recruitment window, a quick setup was essential. However, the mean site setup time was 26 weeks, varying from 15 to 41 weeks (Fig. 4, time taken for sites to open to recruitment or decline participation from initial approach). Reasons for the delays included prolonged (i) decision to participate (sites took between 2 days to 25 weeks before declining the study), (ii) feasibility questionnaire return, (iii) review and sign-off of site agreements and (iv) identification of appropriate site staff.

These delays could be due to factors staff identified as barriers to hospitals taking part, which were not enough time to set up the study, the value of recruiting to studies with low recruitment numbers, lack of consistent support for research associates, strong local and individual ways of treating segmental fractures and concerns about the pragmatic nature of the trial.

The more I think about it, with this short recruitment window, sites start panicking thinking ‘oh no we’ve been in this set up for two months, what are we going to do but it still hasn’t happened, should we bail, should we carry on, how is this going to work’.

Staff 7 (research staff)

I think the concern by the big framing units is that you’re not comparing like with like and so most nails are fairly similar… but comparing a four ring or six ring frame against a TSF [Taylor Spatial Frame] two ring with a couple of half pins is very different and so you’re trying to bundle a load of things into one cohort…I know it’s a feasibility study but the trial was almost too broad, looking at too many different practices.

Staff 10 (surgeon)

iii) Lack of surgical equipoise was a barrier to the feasibility of this study. Treating a segmental tibial fracture was a complex decision that required a thoughtful team approach. Staff expressed that doing the best for patients in the longer term would always take priority. The severity of the injury made STIFF-F much more risky compared to other trials, as there was more that could go wrong.
  I think your eligibility criteria includes open segmental tibial fractures, which are really high risk for amputation, the more risk that you’re asking surgeons to commit to when you’re asking them to randomise a patient, I think the more responsibility they feel and the more uncomfortable they feel with it.
  Staff 6 (surgeon)

Surgeons’ skills and expertise were based on their training and experience. More surgeons were skilled with IMN than CFEF, and preference was often linked to their skill set. In light of these factors, staff felt group equipoise was important, with time to discuss the patient’s needs within a team with a balanced skill set. In this study, opportunities to include patients were missed when a balanced team was not available, for example at weekends.

#### Recovery from a segmental tibial fracture is challenging, and current rehabilitation protocols for patients are inconsistent

Patients and staff identified the long term, life-changing nature of this injury and slow recovery for patients regardless of treatment.

That’s it, yes, a very long process basically. It’s like a year out of your life.

Patient

Staff described differences between the recovery pathways for patients with these interventions and suggested that CFEFs require more rehabilitation support. IMNs were often considered ‘kinder’ for patients as they were internal, while CFEFs were visible and could be seen as disabling, stigmatised and burdensome. For patients with CFEF, the importance of developing confidence with the frame, crucial for weight-bearing activities, skillful pin site care and the difficulty adapting to and living with a frame were also highlighted.

Yes, our frame patients come every single week. They have like a multi-user rehabilitation type clinic where they have a room that looks like a gym in the physio department and they come there. It’s nice for them because they have like a group physio session, where they progress with their physio, they get some physio input, they see the nurse for help with adjusting the frame and any problems they’ve got, and to look at any new infections and whatever.

Staff 6 (research staff)

There are numerous complications with it (CFEF), the adjustment of clothing, wound care, whether someone’s going to be able to walk properly with it, because you can’t put your feet together. Rest and sleeping with it, what happens if the pin sites get infected? There’s a lot of issues with them but I know some doctors really like them because you can fix them without disrupting the fracture site.

Staff 15 (research staff)

##### Rehabilitation survey findings

The findings of the rehabilitation survey revealed that current rehabilitation protocols for segmental tibial fracture patients are limited. Responses indicate that only five of the 11 hospitals (45%) have a rehabilitation programme, protocol or list of recommendations for rehabilitation following IMN and CFEF. In hospital, early post-operative rehabilitation is similar across hospitals and for both interventions. The majority of respondents report that rehabilitation starts most frequently on day 1 postoperatively [8 (73%) for IMN; 10 (91%) for CFEF]. Patients receive weekend physiotherapy based on need, with ten hospitals (91%) providing physiotherapy services every day and one (9%) providing weekday physiotherapy only. Rehabilitation activities are similar for both interventions. All hospitals reported mobility-based rehabilitation activities such as bed exercises, mobility assessment and home circumstance assessment, but only seven reported respiratory-based activities such as breathing exercises and chest check (Table 2, postoperative rehabilitation identified by 11 respondents).

**Table 2:** Post-operative rehabilitation identified by 11 respondents

Aspect of care	IMN ***n*** (%)	CFEF ***n*** (%)
**Wound check**	10 (91)	9 (82)
**Education and advice**	11 (100)	11 (100)
**Bed transfers**	11 (100)	11 (100)
**Breathing exercises**	7 (64)	7 (64)
**Respiratory assessment/chest check**	6 (55)	6 (55)
**Bed exercises**	11 (100)	11 (100)
**Mobility assessment**	11 (100)	11 (100)
**Home circumstance assessment**	11 (100)	11 (100)

**Fig. 5:**
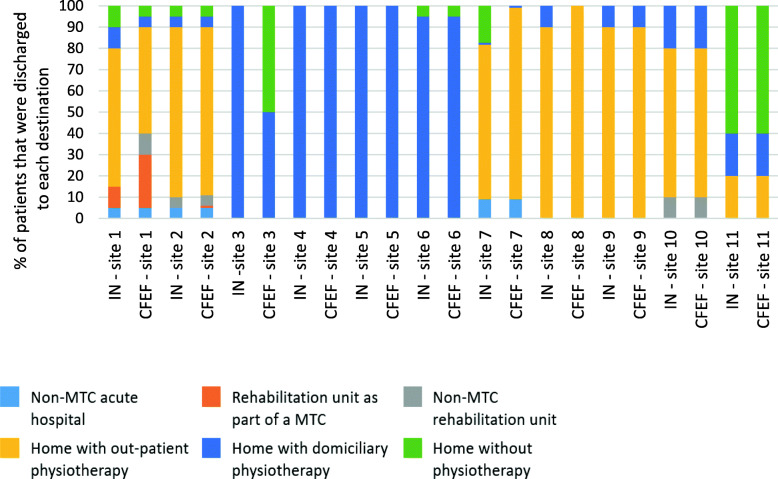
Survey of respondents’ discharge destinations for patients following IMN and CFEF (%)

Discharge destination and planned post-discharge rehabilitation varied by hospital (Fig. 5, survey of respondents’ discharge destinations for patients following IMN and CFEF (%)). Factors that influenced discharge destination are presented in Table 3, factors that influence discharge destination for IMN and CFEF patients from 11 respondents. Most respondents (*n*=10) report that either out-patient physiotherapy or domiciliary physiotherapy are planned for the majority of patients post-discharge. However, in six of the 11 hospitals, respondents reported that between 5 and 60% of patients are discharged home without planned physiotherapy.

**Table 3:** Factors that influence discharge destination for IMN and CFEF patients from 11 respondents

	IMN ***n*** (%)	CFEF ***n*** (%)
**Bed availability in MTC**	1 (9)	1 (9)
**Geographical location of patient’s home**	4 (36)	4 (36)
**Clinical presentation**	9 (82)	9 (82)
**Home circumstance**	11 (100)	11 (100)
**Medical (non-rehabilitation) requirements**	5 (45)	5 (45)
**Other**	1 (9)	2 (18)

#### A way forward

Despite the frustration generated by the study, staff learnt from taking part and felt it was worth the effort.

It is unfortunate, it’s better to have tried and failed than not tried at all and it’s not a failure because we’ve still got information from the trial.

Staff 9 (research staff)

They felt the research question and outcomes such as patient recovery, time to healing and complications continued to have validity. The way forward was identified by participants as (i) alternative study designs to allow for research into rare injuries where an RCT may be unachievable, and (ii) recognition that for this complex injury, surgeons are best placed to decide treatment for each patient within their particular circumstances and the surgeon’s particular skill set.

So I think that’s why it’s better that the surgeon is doing what they want, then that is doing the right thing by the patient…I think it would be quite tough to randomise people in the customary way, but I think it’s not difficult to get a variation of treatment, because there are variations in opinions as to how we manage them, so let people treat them as they wish, but follow the patients appropriately. I think you’d get more information that way than by only randomising three people and not getting a study off the ground.

Staff 12 (surgeon)

## Discussion

This feasibility study adds to the existing evidence of the particular challenges of conducting RCTs of surgical interventions for rare and complex conditions [9–11, 23]. The primary outcome demonstrates that recruitment was much slower than anticipated, and therefore, a full-scale RCT is not feasible. The key findings are that (i) at the participating sites this injury was rare, (ii) the RCT design had limitations due to the complex nature of segmental tibial fractures, and surgeons in particular had strong treatment preferences, which was compounded by administrative delays and lack of support, (iii) there was a contextual difference between the two treatments and recovery pathways, and a lack of protocols for rehabilitation and (iv) the research question is important and alternative study designs should be encouraged for studies of rare complex injuries such as segmental tibial fractures.

Fewer than anticipated patients were screened and recruited at all sites, and staff emphasised the rarity of the injury at interview. Experienced trauma teams used all existing methods which had proved successful in other trauma RCTs to ensure identification of segmental tibial fractures [5, 12]. However, systems for recording accurate numbers of segmental tibial fractures in the UK are poor, and it is possible that patients were missed. In a recent RCT of two surgical interventions for distal femoral fractures, almost half of the distal femoral fractures (47%) seen in the recruiting centres during the recruitment window were not recorded on screening logs [23]. Recruitment was further constrained by long setup periods and a short recruitment window. Drawing on all the MTCs in the UK, a full study would still require a 10-year recruitment period which is untenable.

In addition to rarity and a lack of surgical equipoise as identified within the existing literature [23], the RCT design was problematic in this study due to the complexity of the injury. Segmental tibial fractures are often associated with multiple, complex injuries, with multiple competing needs, limiting the patient’s ability to take part in the study. Surgeons have strong preferences for treatment based on their expertise and experience. The ideal environment for this study was a group decision regarding eligibility and treatment within a balanced team, which includes expertise in both treatments. In practice, this was not always possible, for example, a surgeon familiar with CFEF was not always available at weekends. Challenges in setting up a study of a rare injury and lack of consistent research staff support compounded these issues and created high levels of frustration for staff.

The life changing nature of this injury regardless of the intervention was highlighted by patients and staff. Similar to other fractures (4), recovery was arduous for patients requiring intense physical and psychological work to sustain a momentum and return to daily life. The difference between the two interventions is notable. Additional factors for those with a circular frame included learning to live with the frame, practically, in relation to body image and the anxiety created by pin site care. The rehabilitation survey shows that in 11 hospitals both interventions have planned physiotherapy in hospital, and the majority have planned physiotherapy post discharge, but there is a lack of consistent protocols for rehabilitation. At interview, staff identified the high level of resources required to support patients with circular frames and the variation in resources across NHS Trusts. Further research is needed to clarify current rehabilitation provision for patients with a segmental tibial fracture beyond the 11 hospitals who responded to this survey and to identify what additional support is required for patients living with CFEF.

All participants felt the research question was important. Staff believed it was still important to learn more about patient outcomes after a segmental tibial fracture in order to improve treatment decisions for future patients. However, for this patient group, evidence is required that derives from research designs which allow for complexity in relation to patient characteristics, the injury and service provision.

### Strengths and Limitations

This study used mixed-methods to assess the feasibility of a RCT comparing IMN and CFEF for segmental tibial fractures. It included a multicentre pilot RCT, interviews with staff and patients and a survey of physiotherapists. Interviews with patients and staff provided an understanding of their experience of STIFF-F and the issues raised by the pilot RCT. Due to the low number of patients screened and recruited, some of the secondary objectives of this study were not met. Lack of patients meant the qualitative patient data was not saturated and further research is needed to explore this aspect of the study. The limited number of centres responding to the rehabilitation survey limits the utility of this data.

## Conclusion

RCTs of rare and complex orthopaedic injuries can face insurmountable challenges. In this study of segmental tibial fractures, complexity in relation to the patient, injury, treatment and recovery was identified as barriers to inclusion in a RCT.

## Data Availability

No further supplementary material is included.

## Abbreviations

STFs: Segmental tibial fractures
IMN: Intramedullary nailing
CFEF: Circular frame external fixation
RCT: Randomised controlled trial
MTCs: Major Trauma Centres
DRI: Disability Rating Index
PSQI: Pittsburg Sleep Quality Index
TSK: Tampa Scale for Kinesiophobia
EQ-5D-5L: European Quality of Life score
RUST: Radiographic union scale in tibial fractures
OCTRU: Oxford Clinical Trials Research Unit
