# Correction to: Reamed intramedullary nailing versus circular frame external fixation for segmental tibial fractures (STIFF-F): a mixed methods feasibility study

**DOI:** 10.1186/s40814-021-00842-y

**Published:** 2021-04-30

**Authors:** Caroline B. Hing, Elizabeth Tutton, Toby O. Smith, Molly Glaze, Jamie R. Stokes, Jonathan Cook, Melina Dritsaki, Emma Phelps, Cushla Cooper, Alex Trompeter, Michael Pearse, Michael Law, Matthew L. Costa

**Affiliations:** 1 Department of Trauma and Orthopaedics, St George’s University Hospitals NHS Foundation Trust, London, UK; 2 Nuffield Department of Orthopaedics, Rheumatology and Musculoskeletal Sciences, University of Oxford, Oxford, UK; 3 Faculty of Medicine and Health Sciences, University of East Anglia, Norwich, Norfolk UK; 4 Imperial College London, London, UK

**Correction to: Pilot Feasibility Stud 7, 93 (2021)**

**https://doi.org/10.1186/s40814-021-00821-3**

Following publication of the original article [1], the authors reported an error in the fifth author’s last name. The fifth author should be *Jamie R. Stokes* instead of Jamie R. Law.

The original article has been corrected.
